# Effect of Bayer Red Mud on the Mechanical Strength of Grouting Material

**DOI:** 10.3390/ma18040788

**Published:** 2025-02-11

**Authors:** Xiran Li, Yanna Han, Guorui Feng, Jinwen Bai, Junbiao Ma, Guowei Wu, Shengyu Su, Jiahui Qiu, Mingzhuang Lv

**Affiliations:** 1 College of Mining Engineering, Taiyuan University of Technology, Taiyuan 030024, China; lixiran1228@link.tyut.edu.cn (X.L.);; 2 Key Laboratory of Shanxi Province for Mine Rock Strata Control and Disaster Prevention, Taiyuan University of Technology, Taiyuan 030024, China; 3 Shanxi-Zheda Institute of Advanced Materials and Chemical Engineering, Taiyuan 030024, China; 4 Shanxi Research Institute for Clean Energy, Tsinghua University, Taiyuan 030024, China

**Keywords:** Bayer red mud, grouting material, early strength, hydration reaction, synergistic effect

## Abstract

The massive stockpiles of Bayer-process red mud (BRM) severely compromise soil integrity, necessitating the urgent development of efficient large-scale utilization strategies. BRM contains large amounts of calcium, silicon, and aluminum. Theoretically, water glass and flue gas desulfurization gypsum (FGD) can increase the active substances in BRM, making it a cementitious raw material capable of replacing cement. This study pioneers a novel activation strategy utilizing water glass–FGD synergism to amplify the BRM reactivity, enabling an increased dosage in construction materials through enhanced pozzolanic activity. They were blended into the cement at different ratios to prepare a grouting material (BF-C) for fissure sealing in mine rock strata. The hydration mechanism of BF-C was analyzed from a micro perspective by XRD, FTIR, ICP-OES, and SEM-EDS, and combined with the Ca/(Si + Al) ratio to reveal its hydration synergy. The results showed that the 3 d and 28 d strength of 70% BRM-FGD reached 8.94 MPa and 13.71 MPa, respectively. At this ratio, the hydration synergy of BF-C was the strongest. The addition of water glass and FGD can directly modulate the Ca/(Si + Al) ratio of the system to an optimal value of 0.94, which promotes the formation of early hydration products. C-S-H gel, calcite, and C(N)-A-S-H are the main hydration products of BF-C. C-S-H gels are encapsulated on cancrinite, and their three-dimensional network structures are dense. Meanwhile, C(N)-A-S-H crystals are interspersed between C-S-H gels, making the structure more stable. This achievement introduces an innovative method for the large-scale utilization of Bayer red mud, providing an effective solution in grouting technology using solid waste as raw material.

## 1. Introduction

Grouting technology has become one of the main methods adopted for underground engineering disaster prevention and control [1]. As the core of grouting technology, the characteristics of grouting materials, including fluidity and mechanical properties, directly determine the successful implementation of the project [2,3]. To prevent delaying the project progress, the grouting material is usually required to have an effective bearing capacity within a short time, implying the need for a high early strength. Researchers have improved the early strength of materials in two main ways: one is by modifying cementitious raw materials [4,5,6], and the other is by adding admixtures that increase the hydration reaction rate [7]. However, the use of traditional cement as the main grouting material in recent years has led to a series of associated problems, including energy consumption, increasing demand for raw materials, and the greenhouse effect [8,9,10]. Therefore, finding alternative materials has become an urgent need. The resources sector and energy industries, such as ore mining and metal smelting, have generated large amounts of solid wastes. These solid wastes have potential reactivity but need to be artificially stimulated. The conversion of solid wastes from the industrial and mining sectors into new environmentally friendly materials that can replace cement through technological innovations not only reduces the amount of cement used but also reduces the accumulation of solid wastes, which has become the focus of research.

Bayer red mud is the main solid waste generated in the process of the industrial production of alumina. It appears reddish brown in color due to its high content of iron oxide. The Bayer process adds an NaOH solution to the crushed bauxite to precipitate aluminum hydroxide. Therefore, Bayer red mud obtained through this method contains a high level of Na_2_O, which is the primary reason for its strong alkalinity. According to statistics, the current global stockpile of red mud has exceeded 2.7 billion tons, and is still discharged in the amount of 120 million tons per year [11]. Bayer red mud has a fine particle size. Its particle diameter ranges from 0.088 to 0.25 mm. The pH value of its leaching solution is in the range of 12.1 to 13.0. Its specific gravity is 2.7~2.9, and its unit weight is 0.8~1.0 g/cm^3^. It has a high water-holding capacity [12]. Some physical properties of red mud are listed in Table 1. Bayer red mud is mainly composed of Fe_2_O_3_, Al_2_O_3_, SiO_2_, Na_2_O, and CaO, and also contains small amounts of TiO_2_, SO_3_, K_2_O, and MgO [13,14,15,16,17,18]. Because Bayer red mud contains calcium, silicon, and aluminum, using it to prepare building materials is an effective way to achieve the large-scale consumption of Bayer red mud [19,20,21]. Further, the study by Xiao et al. [22] has shown that the early-stage strength of magnesium phosphate cement mortars can be improved by 14.8% compared with the control group when an appropriate amount of red mud (15 wt%) is added. The active substances in red mud during the hydration reaction are Fe_2_O_3_, Al_2_O_3_, and CaO. Kang et al. [23] noted that the cement paste with neutralized liquefied red mud developed strength earlier. Li et al. [3] found that the addition of red mud prolonged the initial setting time of the grouting material from 2.5 h to 5 h and the final setting time from 5.3 h to 7.6 h. The viscosity was reduced from about 2400 mPa×s to about 1000 mPa×s when 70 wt% of red mud was added, and, after physical grinding, the red mud enhanced the stability of the slurry. Zhang et al. [24] showed that mixing 60 wt% red mud with the mean particle size of 392 μm with slag resulted in a slurry with a flow diameter of about 26.8 cm. Red mud was not only one of the reactants, but also had a filling effect, causing the material to have a low porosity. Thus, the addition of red mud to the cementitious raw materials can effectively improve the early strength of the grouting materials and bring considerable economic and environmental benefits [25,26].

For the preparation of early-strength grouting materials using red mud, it is necessary to regulate the Ca/(Si + Al) ratio and adopt appropriate activation methods. Alkali excitation, mechanical grinding, and high-temperature heating are the main activation methods for enhancing the reactivity of solid wastes generated in various industries. Essentially, they increase the content of active substances in these wastes [27,28,29]. Among these methods, alkali excitation is the most common and low-carbon activation approach for mineral solid wastes compared to mechanical grinding and high-temperature heating. The main alkaline activators commonly used are sodium silicate solution (water glass solution) and NaOH solution [10,30,31,32]. Kim et al. [33] discovered that NaOH is highly effective in increasing fluidity. More importantly, it significantly improves the degree of reaction of the system, resulting in an increased formation of calcium silicate hydrates (C-S-H) and enhancing the early strength of the blended system consisting of red mud, Ca(OH)₂, Na₂CO_3_, and fly ash. In addition, the mechanical properties of the grouting material are directly affected by SiO_2_/Na_2_O molar ratios and alkaline activator concentrations [34]. Davidovits [35] reported that the molar ratio of SiO_2_/M_2_O for a user-friendly alkaline reagent should be greater than 1.65. Geng et al. [13] used this as a reference to study the effect of different factors on the compressive strength of materials. Considering the high cost of water glass, its mixed dosage should be minimized. The work of Wang et al. [16] clearly demonstrated that the degree of polymerization of the hydration products is affected by the Ca/(Si + Al) ratio. Moreover, the properties of the material system can be significantly influenced by changes in this ratio. In their research, when the Ca/(Si + Al) ratio was 0.96, the eco-friendly revetment block had the highest degree of polymerization. Therefore, through the dissolution of the reactive calcium, silicon, and aluminum components of the red mud by alkali excitation, the Ca/(Si + Al) ratio is altered, and the reactive products of the red mud are affected. Thus, we are expected to achieve early strength properties of red-mud-based cementitious grouting materials.

Flue gas desulfurization gypsum (FGD) is a by-product of the process of using lime-limestone to remove sulfur dioxide from flue gases of coal or oil combustion, and the main chemical composition is calcium sulfate dihydrate (CaSO_4_·2H_2_O). In previous years, scholars investigated the performance of FGD as a cementitious raw material [36]. Li et al. [15] found that the presence of SO_4_^2−^ accelerates the dissolution of Al^3+^ and Si^4+^ from the red mud. Since FGD contains Ca^2+^, the addition of FGD can enhance the properties of red-mud-based grout. The results of Liu et al. [37] indicated that the addition of FGD can effectively activate the cementitious activity of red mud, which significantly increases the compressive strength of concrete. In theory, FGD can be used as a solid-waste-type activator to excite the activity of red mud together with water glass and promote the early compressive strength of the grouting materials.

Some progress has been made in the research on BRM. However, due to the high cost of disposal and the low economic returns, most aluminum producers still choose to dispose of their aluminum waste (BRM) in local landfills. This is convincing evidence that the industry lacks an operationally simple and cost-effective method for red mud processing, which is also the research gap in the field of red mud. This research originally proposed a method to effectively enhance the BRM activity by mixing a solid waste, flue gas desulphurization gypsum (FGD), with water glass. BRM and FGD were mixed with a small amount of cement to prepare a grouting material. The promotion of the hydration reaction by the Ca/(Si + Al) ratio in the BRM gelling system was researched though the comparison of compressive strength. The synergistic effect and hydration mechanism among BRM, FGD, and cement were analyzed from a micro perspective. The purpose of this work is to increase the utilization rates of BRM and FGD, maximize cement replacement, and ensure that the workability and strength of the grouting material meet the standards. This study provides a new idea for the large-scale utilization of Bayer red mud.

## 2. Materials and Methods

### 2.1. Materials

#### 2.1.1. BRM

**Figure 1:**
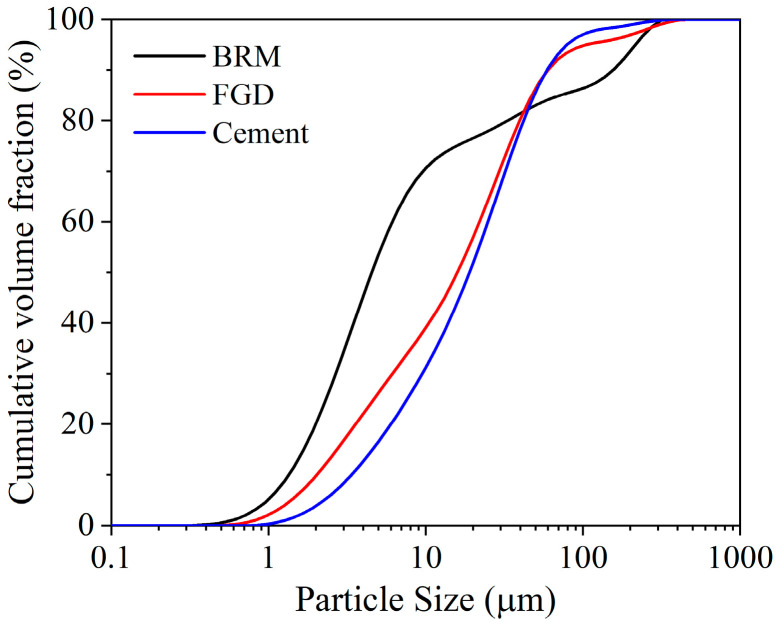
Particle size distributions of BRM, FGD, and cement.

Bayer red mud (BRM), provided by a coal-fired power plant and aluminum production company in Shanxi Province, China, was used in the experiment. The untreated BRM is a dark red, water-saturated block. According to the standard *Methods for testing uniformity of concrete admixture* (GB/T 8077-2012, 2012) [38], the raw BRM was thoroughly mixed with water to prepare a saturated solution. Then, the filtrate was taken for pH determination using a pH meter. The pH of the filtrate was 9.08. As a pretreatment process, the raw BRM material was broken into small pieces less than 2 cm in size and dried under natural conditions for 5 days. Afterward, it was crushed and ground by a ball mill until the sample could pass through a sieve with a mesh size of 0.425 mm (35 mesh screen). Then, a vacuum drying oven was used to heat the sample at 60 °C for 6 h. After heating, the sample was cooled down to room temperature. As the BRM samples have high water absorbency and are prone to easy agglomeration of particles, after the above steps, it is necessary to store the processed samples in a desiccator. The particle size distribution of BRM is shown in Figure 1, which was obtained using a Mastersizer 2000 (Malvern Panalytical Ltd., Malvern, UK). The specific surface area of BRM is 1910 m^2^/kg.

**Figure 2:**
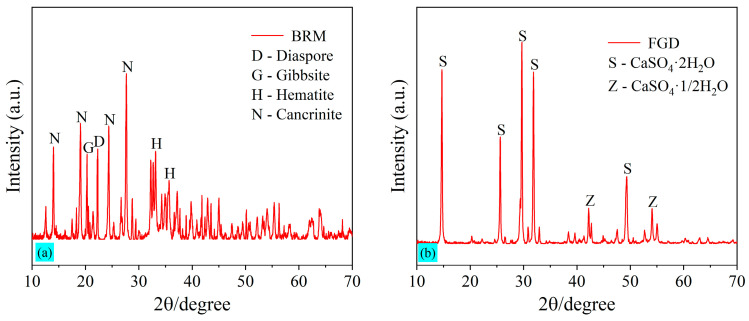
XRD pattern of the raw materials: (**a**) BRM and (**b**) FGD.

Table 2 summarizes the chemical compositions of the selected raw BRM, as obtained by a Zetium X-ray fluorescence spectrometer (i.e., XRF) produced from Malvern Panalytical. Powdered samples of raw materials are tableted before being placed in the testing equipment. The boric acid inlay matting method of sample preparation was used. The main components of BRM are aluminum oxide (Al_2_O_3_), silicon dioxide (SiO_2_), ferric oxide (Fe_2_O_3_), and calcium oxide (CaO), accounting for 74.35% of the total chemical composition. The XRD spectrum of the raw BRM is shown in Figure 2a. The mineral compositions of BRM are complex, and most of them exist in various crystalline forms. Hematite (Fe_2_O_3_), Cancrinite [Na_6_Ca_2_Al_6_Si_6_O_24_(CO_3_)_2_·2H_2_O], Diaspore [AlO(OH)], and Gibbsite [Al(OH)_3_] are the main mineral compositions of the BRM.

#### 2.1.2. FGD

Flue gas desulfurization gypsum (FGD) in the study was purchased from Shanxi Youlaibo Chemical Technology Co., Ltd., Taiyuan, China. The chemical compositions of the purchased FGD determined by XRF are summarized in Table 2, and it can be seen that the purity of FGD is relatively high, and the total content of CaO and SO_3_ is 94.16%. As is the case with the XRF test result, the XRD pattern of FGD in Figure 2b also shows that the mineral compositions of FGD are mainly calcium sulfate dihydrate (CaSO_4_·2H_2_O) and calcium sulfate hemihydrate (CaSO_4_·1/2H_2_O). Because the FGD is originally in dry powder form, no crushing, grinding, or drying steps are required. The pretreatment process of FGD is similar to that of BRM, and the sample is able to pass through a sieve with a mesh size of 0.425 mm. The particle size distribution of FGD is shown in Figure 1, and the specific surface area of FGD is 1050 m^2^/kg.

#### 2.1.3. Cement

The cement selected in the study was 42.5-grade ordinary Portland cement (P.O.). It was purchased from Shanxi Youlaibo Chemical Technology Co., Ltd. Its chemical compositions determined by XRF are summarized in Table 2. Its 3 d, 7 d, and 28 d compressive strengths were tested to be 14.82 MPa, 21.12 MPa, and 35.62 MPa, respectively. The initial setting time was 187 min and the final setting time was 574 min. The stability of this cement was determined by Ray’s clamp method and the average value of expansion measured was <5.0 mm. In conclusion, all the properties of the cement selected in this study complied with the standard *Common Portland cement* (GB 175-2020) [39].

#### 2.1.4. Alkaline Activator

In the experiment, the final alkaline activator was a 1.65 M (Na_2_O·1.65SiO_2_) water glass, which was mainly composed of the sodium silicate raw liquid with a modulus of 3.3 and a concentration of 34%. The two activators, sodium silicate and sodium hydroxide (NaOH solid particles), were purchased from the same supplier. The modulus of the water glass was adjusted by the addition of NaOH solid particles. For example, 100 g of water glass raw liquid (3.3 M) contains 25.89 g of SiO_2_ and 8.11 g of Na_2_O. As a result, if its modulus is adjusted to 1.65 M, 10.46 g of the NaOH solid particles need to be added to the 3.3 M water glass raw liquid per 100 g.

### 2.2. Methods

#### 2.2.1. Mixture Proportion Design and Sample Preparation

Adding the FGD is beneficial for improving the activity of BRM. However, excessive FGD will cause the materials to solidify too rapidly and release a large amount of heat [15]. According to the preliminary experiments and research by others [40], the prepared materials demonstrated good performance when the ratio of BRM to FGD was 19:1. Keeping this ratio constant in the subsequent research, the effects of BRM on workability and mechanical properties of the materials were studied by changing the cement content. The mass ratios of the BRM-FGD-Cement (BF-C) grouting materials were determined as BRM: FGD: cement = 95:5:0, 85.5:4.5:10, 76:4:20, 66.5:3.5:30, 57:3:40, and 0:0:100, corresponding to samples BF100C0, BF90C10, BF80C20, BF70C30, BF60C40, and BF0C100, respectively, as shown in Table 3. In addition, the dosages of the alkaline activator (diluted by water, containing 4.45% Na_2_O, 7.14% SiO_2_, and 88.41% H_2_O) and the water reducer (Naphthalene) were also determined, and the water/solid ratio was 0.5.

**Figure 3:**
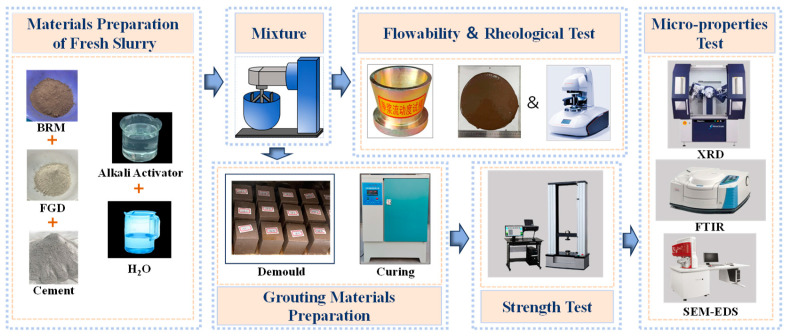
The preparation process of BF-C grouting material.

The detailed preparation process of the sample materials is illustrated in Figure 3. The dry BRM, FGD, and cement powders were uniformly mixed with the alkaline activator in a mixer for 5 min. After thorough mixing, the freshly prepared slurry was poured into the plastic cubic molds measuring 40 mm × 40 mm × 40 mm. Subsequently, the molds were vibrated to achieve good compaction. The molds containing the slurry were then placed for curing at room temperature for 24 h. Afterward, the solidified samples were removed from the molds and placed in a standard curing box with constant temperature and humidity (20 ± 1 °C, 90 ± 5% RH) until reaching the predetermined curing times of 3, 7, and 28 days.

#### 2.2.2. Test Methods of Fluidity and Rheological Characteristics

The fluidity of the slurry was measured in accordance with GB/T 8077-2012 [38]. In addition, another fluidity index of the slurry is its rheological characteristics, which were obtained by using an MCR-72 torque rheometer (Shanghai Anton Paar Trading Co., Ltd., Shanghai, China). The fresh slurry was evenly mixed with a shear rate of 100 s^−1^ for 30 s; subsequently, the rheometer was set to reduce the rate from 100 s^−1^ to 0 s^−1^ within 300 s. The experiments were repeated twice to ensure the reproducibility of the data.

#### 2.2.3. Equilibrium Moisture Content

In order to better analyze the law of influence of slurry fluidity, the equilibrium moisture content (EMC) was measured by the desiccator method [41,42]. Samples with a cement-to-BRM-FGD ratio of 19:1 were placed in a bucket that could be closed with a lid, and then were equilibrated for two weeks in desiccators at 30 °C in a thermostatted cabinet. This equilibration process was conducted in the presence of saturated solutions of various salts to control the relative humidity [43]. Table 4 lists the salt solutions used [44]. After equilibration, the samples were dried in a vacuum oven for 24 h at 30 °C. The EMC was calculated by Equation (1) [43].

(1) $$EMC=\frac{m_{2}-m_{1}}{m_{1}-m_{0}}\times 100\%$$

where *m*_0_ is the weight of the dish, g; *m*_1_ is the weight of the dish and sample after drying, g; and *m*_2_ is the weight of the dish and sample after equilibration, g.

#### 2.2.4. Uniaxial Compressive Strength

The 40 mm × 40 mm × 40 mm cubic samples, having been cured to the designed ages (3 d, 7 d, and 28 d), were removed from the curing box, and we immediately measured the uniaxial compressive strength by an electro-hydraulic servo press. When the real-time pressure value reached 0.1 kN, it indicated the end of the first stage. Then, the second stage commenced with a loading rate of 0.4 mm/min controlled by the displacement. Three samples were tested in each group and the average value was calculated as the final data. To ensure test accuracy and stability, the sample surfaces were leveled during casting to eliminate stress concentrations from end-contact defects and leveled before loading to prevent eccentric loading.

The predicted value of the compressive strength of grouting materials is calculated by the following equation, Equation (2).

(2) $$P_{\mathrm{BFxCy}}=M_{\mathrm{BF}100C0}\times x\;\%+C\times y\;\%$$

where *M*_BF100C0_ means the measured value of the compressive strength of sample BF100C0, *x*% means the percentage of BRM-FGD in raw materials, *C* means the compressive strength of the tested sample BF0C100, and *y*% means the percentage of cement in raw materials.

#### 2.2.5. XRD, FTIR, and ICP-OES

After the compression test, the residual samples were stored in ethanol for 3 days to prevent further hydration. Before the microstructural analyses (e.g., XRD and FTIR), the samples were dried at 60 °C for 2 h in a vacuum-drying oven and then ground to pass through a sieve with a size of 0.075 mm. The XRD pattern was obtained by a Rigaku SmartLab X-ray diffraction meter manufactured in Japan, which was using Cu Kα radiation and operated with 2θ ranging from 10° to 80° at 10°/min. The XRD pattern of raw material in Figure 2 was also obtained by the same procedure. Moreover, the infrared radiation (IR) data were acquired by a Thermo Scientific Nicolet FTIR spectrometer produced by Thermo Fisher Scientific Inc., Waltham, MA, USA, and the samples were measured within a range of 400–4000 cm^−1^. The above-measured data were corrected according to a baseline and normalized for comparison and analysis.

For further analysis, the active components in BRM and FGD were determined by using an Inductively Coupled Plasma Optical Emission Spectrometer (ICP-OES). The powder samples of BRM and FGD were dissolved in an alkaline solution in advance. The determination was carried out using an Agilent ICP-OES 725 ES model.

#### 2.2.6. SEM-EDS

Some block-shaped residual samples were selected after the compression test. The microscopic morphology of these samples was investigated by scanning electron microscopy (SEM, TESCAN MIRA LMS) equipped with energy-dispersive X-ray spectroscopy (EDS). The examined samples were small pieces approximately 10 mm in size and had a flat surface. Since the hydration stoppage was required for the SEM-EDS test, the samples were firstly stored in ethanol and then dried in the vacuum drying oven at 60 °C for 2 h. In order to be sure that the results obtained were representative of the entire material, multiple samples from each set of ratios were selected at different locations and were analyzed by SEM-EDS.

## 3. Results and Discussion

### 3.1. Fluidity and Rheological Characteristics

**Figure 4:**
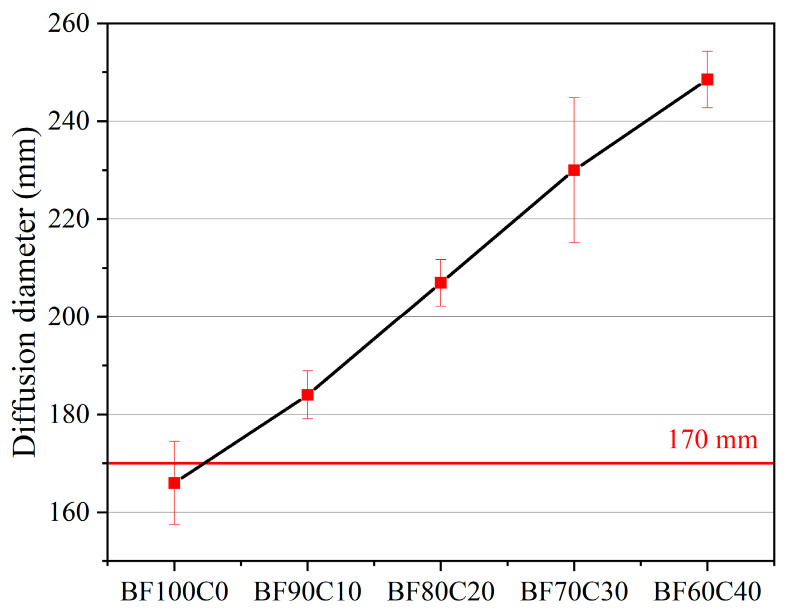
Effect of BRM-FGD content on fluidity of the slurry.

The fluidity test data of the grouting material slurry is shown in Figure 4. From this, it can be obtained that the fluidity of the slurry is enhanced with the decrease in the BRM-FGD content. The incorporation of cement can improve the fluidity of the slurry. In Figure 1, the particle size distribution of BRM is smaller than that of cement. However, the water demand of the small-particle-size red mud is high and it has a strong tendency to agglomerate [3,45]. As the raw materials slurry is continuously mixed, a part of the small-particle-size red mud agglomerates, which consequently leads to a decrease in the slurry fluidity upon an increase in red mud.

Referring to the local standard *Technical Guidelines for Construction of CFB Fly Ash and Bottom Ash Grouting and Filling Mined-out Area* (DB14/T 2120-2020, 2020) in Shanxi, China, the fluidity requirement for industrial solid waste grouting material is ≥170 mm [46]. In this study, it is used as a standard for measuring whether the slurry fluidity of BF-C grouting materials meets the grouting requirement. Figure 4 shows that the diffusion diameter of BF100C0 slurry is 166 mm, which is non-compliant. When the BRM-FGD content is less than 90 wt%, all the diffusion diameters of the slurry are greater than 170 mm, which complies with the local standard, with the diffusion diameters of BF90C10, BF80C20, BF70C30, and BF60C40 being 184 mm, 207 mm, 230 mm, and 248.5 mm, respectively.

**Figure 5:**
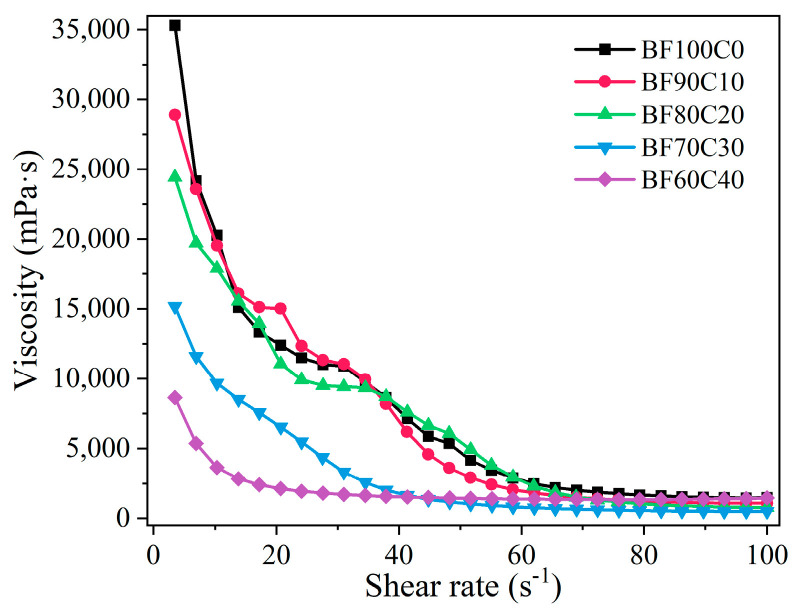
Effect of BRM-FGD content on steady rheological characteristics of slurry.

**Figure 6:**
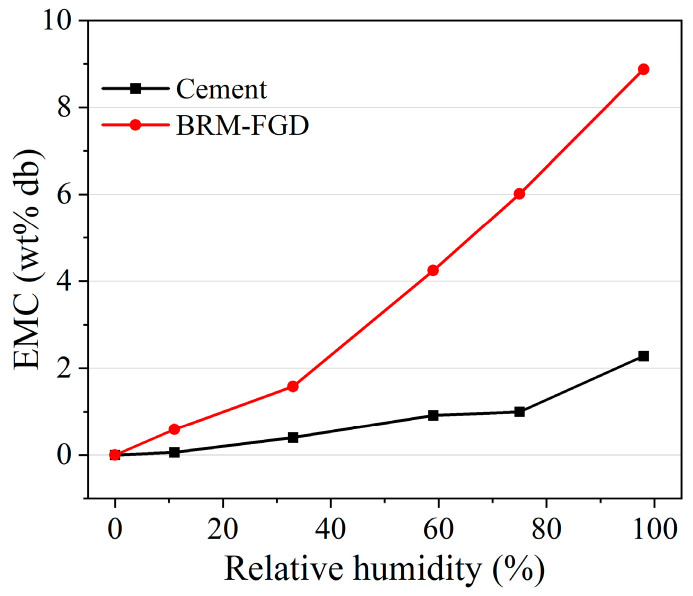
EMC results of different raw materials.

The rheological characteristics of grouting materials determine their pumping distance. This pumping distance is mainly determined by the particle characteristics and hydration process of the raw materials [47]. The effect of the BRM-FGD content on the rheological properties of the BF-C grouting material is shown in Figure 5. It can be observed that, as the red mud content increases, the viscosity of the slurry increases, while the fluidity of the slurry decreases. This is consistent with the result indicated by the diffusion diameter in Figure 4. The calculated EMC results of the raw materials are presented in Figure 6. For comparison purposes, the raw material tested here is a well-mixed blend of BRM and FGD in a ratio of 19:1. As shown in Figure 6, the EMC values of raw cement were lower than those of BRM-FGD, which indicates that BRM-FGD is more absorbent than cement. BRM particles absorb water due to their agglomeration ability, which reduces the free water content in the slurry system and is one of the reasons for the increase in viscosity and decrease in fluidity [3].

Furthermore, the viscosities of slurries for all mixing ratios show a decreasing trend with an increasing shear rate, indicating that the slurries have a shear thinning characteristic. It can also be observed from Figure 5 that the initial viscosities of BF70C30 and BF60C40 slurries are significantly lower compared to those of the first three ratios. From the perspective of the raw material particle characteristics, since the cement particles within the raw material system are interposed between the BRM particles with a small particle size, this prevents the agglomeration of BRM and makes the overall particle gradation of the slurry more reasonable when the mixing ratio of BRM: FGD: cement is 66.5:3.5:30 (i.e., sample BF70C30) [3].

### 3.2. Mechanical Properties

**Figure 7:**
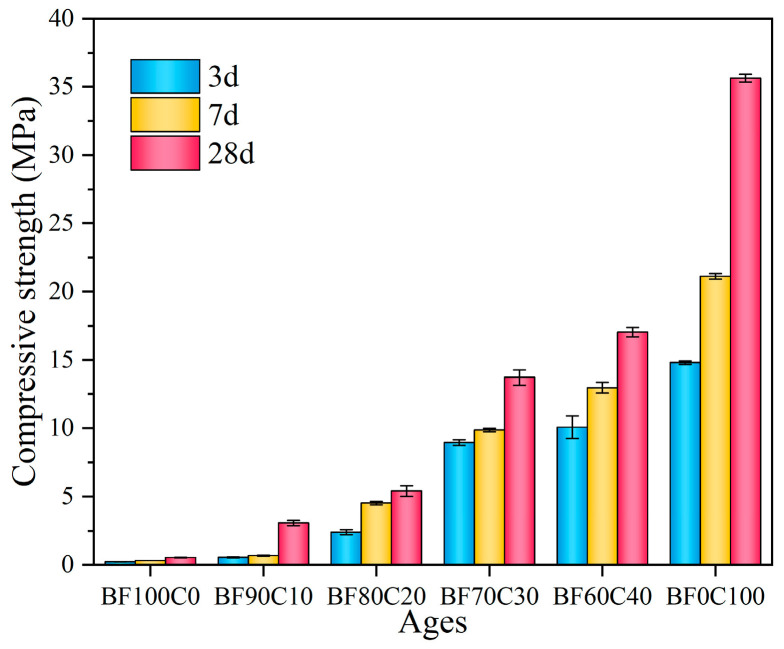
Compressive strength of the samples.

The uniaxial compressive strength of the grouting material is one of the most basic and crucial indicators for determining whether it can be used in a project. And the compressive strength is enhanced with the increase in hydration products. The effect of the content ratio of the BRM-FGD and cement on compressive strength is shown in Figure 7. The uniaxial compressive strength of the materials increased as the curing period (3 d, 7 d, and 28 d) prolonged for all mixing ratios. When the BRM-FGD content was 100 wt%, the uniaxial compressive strength at 3 d, 7 d, and 28 d were 0.23 MPa, 0.30 MPa, and 0.54 MPa, respectively. This indicates that it is effective in obtaining some strength for the grouting material made from low-activity BRM by using the mixture of FGD and water glass as an alkaline activator to stimulate the activity of red mud. However, the stimulating effect is limited [15]. This is because the chemical compositions of BRM mainly include CaO, SiO_2_, and Al_2_O_3_, but most of them exist in crystalline forms such as cancrinite and do not participate in the curing reaction [18]. This is also confirmed by the XRD pattern of the raw red mud in Figure 2a. To further confirm this argument, the active fractions of the BRM and FGD raw materials were determined by ICP-OES, as shown in Table 5. By comparing Table 2 with Table 5, it can be observed that the active Ca content in BRM is merely 1.75%, the active Mg content is 0.23%, the active Al content is 6.86%, and the active Si content is higher at 15.02%. This indicates that BRM, as a whole, has lower activity. Although the active Ca content in FGD is high, its proportion in the overall mixture is small. Therefore, in the absence of a cement addition, the overall slurry system exhibits lower activity and correspondingly lower strength.

Upon mixing the BRM-FGD content with cement, the uniaxial compressive strength increased as the BRM-FGD content decreased (and as the cement content increased). The 3 d compressive strength continued to grow as the BRM-FGD content decreased from 100 wt% to 60 wt%. The values were 0.23 MPa, 0.54 MPa, 2.40 MPa, 8.94 MPa, and 10.06 MPa, respectively. Correspondingly, the compressive strength growth per 10% decrease in BRM-FGD was 0.31 MPa, 1.86 MPa, 6.54 MPa, and 1.12 MPa, respectively. These values are listed more clearly in Table 6. The values of compressive strength with its increment at 7 d and 28 d were also presented in the table, which showed that the increments of sample BF70C30 were the highest compared to the values of other samples. A significant leap in the growth of the 3 d compressive strength was clearly observed when the BRM-FGD content decreased from 80 wt% to 70 wt%, which indicates that the hydration reaction synergistic effect shows the best performance at a BRM-FGD content of 70 wt%, as the strength growth of sample BF60C40 slows down again. This may be due to the fact that the dispersion of the raw material particles has been optimized when the ratio of BRM: FGD: cement is 66.5:3.5:30.

The Ca/(Si + Al) ratio of raw materials is an important parameter for controlling production. For calcium silicate substances, it is one of the main factors affecting their hydraulicity. The addition of water glass and FGD can directly modulate the Ca/(Si + Al) ratio of the sample to an optimal value. The calculated Ca/(Si + Al) ratios for each proportion of samples are listed in Table 3, which shows that the Ca/(Si + Al) ratios of the samples increased with the decrease in BRM-FGD content. In addition, sample BF70C30 is in the range of the intermediate-calcium-based cementitious materials (Ca/Si is 0.6–1.5). Moreover, compared with the high-calcium-based Portland cement (Ca/Si is generally greater than 2) and the low-calcium-based geopolymer (Ca/Si is generally less than 0.5), intermediate-calcium-based cementitious materials are better able to stimulate the original characteristics of solid wastes [16,48,49]. BRM, which is a highly alkaline solid waste, and cement, which is also alkaline, work together in a way whereby the addition of BRM further promotes the dissolution of active substances. Meanwhile, FGD generates more Ca^2+^ and they exhibit a synergistic effect in the slurry system [15,50]. In this study, based on the data of the early compressive strength, sample BF70C30 had a stronger synergistic effect and promoted the early hydration reaction at a Ca/(Si + Al) ratio of 0.94.

Although a higher cement content generally leads to a higher uniaxial compressive strength of the grouting material, considering the economic and environmental aspects, a ratio of BRM: FGD: cement of 66.5:3.5:30 is recognized as the optimum ratio for this study.

**Figure 8:**
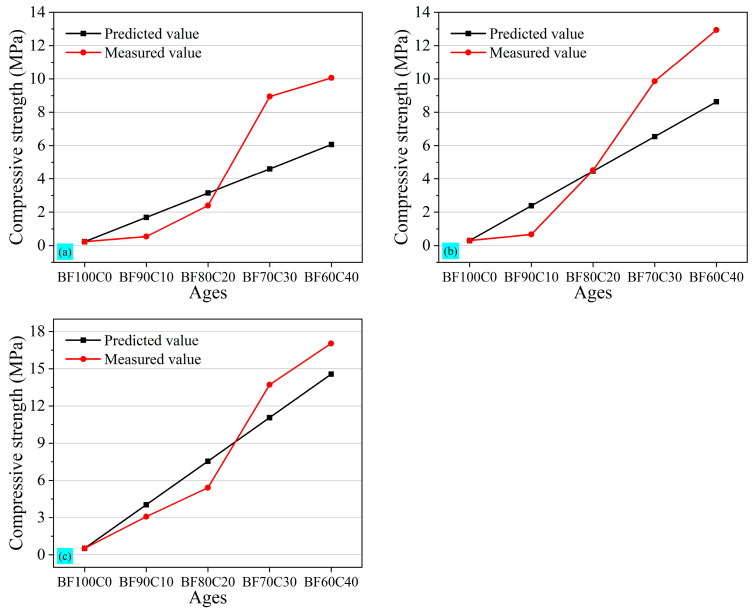
Comparative graphs of (**a**) 3 d, (**b**) 7 d, and (**c**) 28 d predicted and measured compressive strength value.

According to BF0C100, the 3 d, 7 d, and 28 d compressive strength of the general-purpose silicate cement used in this study is 14.82 MPa, 21.12 MPa, and 35.62 MPa, respectively. Based on the compressive strength of the tested samples BF100C0 and BF0C100, the predicted value of the compressive strength of grouting materials was calculated and compared with the measured value. The results were shown in Figure 8. When the BRM-FGD content exceeded 80 wt%, the measured values of the 3 d, 7 d, and 28 d compressive strength were lower than the predicted values. This indicated that the 20 wt% cement added failed to provide the desired strength enhancement.

When the BRM-FGD content was reduced to 70 wt% (i.e., sample BF70C30), the measured values of the 3 d compressive strength were significantly higher than the predicted values; however, the gap between the predicted and measured values decreased from 7 d to 28 d. But the measured value at 28 d was close to the predicted value. This indicated that the hydration reaction proceeded earlier and the early strength of the materials was enhanced. On the one hand, in addition to exciting the activity of BRM, water glass is also an accelerator commonly used in cementitious materials. The silicic acid generated from its hydrolysis can react with calcium-containing raw materials to form calcium silicate, which disrupts the balance of cement hydrolysis and promotes the reaction to proceed in the right direction, forming a large number of C-S-H gels within a short period and thus generating early strength [51]. On the other hand, comparing samples BF80C20 and BF70C30 with the same water glass modulus or dosage, it can be seen that, when the ratio between BRM-FGD and cement was maintained at 70:30, the red mud within the slurry system neither impeded the formation of hydration products in cement nor disrupted their bonding. Furthermore, this ratio facilitated the enhanced leaching of activated silica and calcium components in the red mud themselves, promoting the reaction to proceed in the right direction and leading to the formation of calcium silicate. Considering the lowest content of cement and conforming to the predicted values of compressive strength, the optimum ratio of BRM: FGD: cement is 66.5:3.5:30; this comparative result corresponds to the change in the above-mentioned uniaxial compressive strength.

### 3.3. Micro-Properties of BF-C

**Figure 9:**
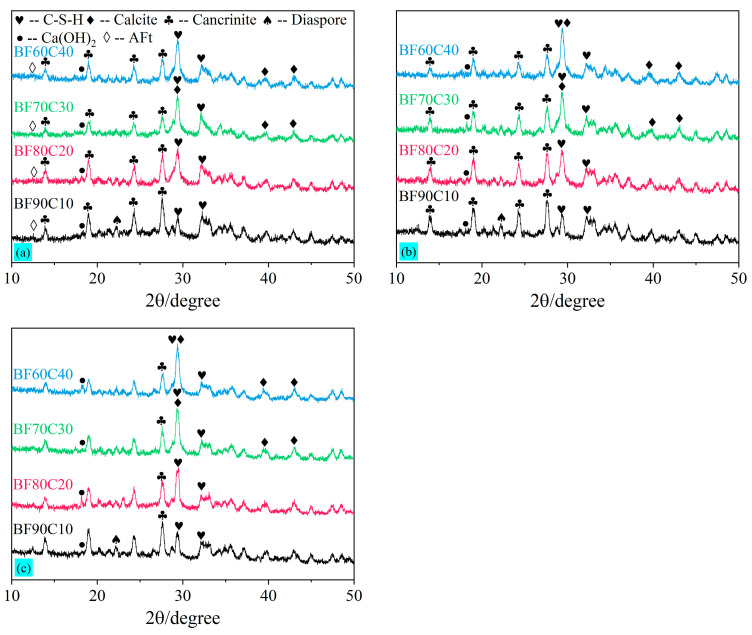
XRD patterns of BF-C grouting materials at (**a**) 3 d, (**b**) 7 d, and (**c**) 28 d.

The XRD test results for the hydration products of BF-C grouting materials at 3 d, 7 d, and 28 d for different ratios are shown in Figure 9. It can be seen that the main hydration products of the BF-C grouting materials are calcium silicate hydrate (i.e., CaO·SiO_2_·nH_2_O, and C-S-H gels), and its main peak is located at 29.062°. In addition, the peaks of Ca(OH)_2_ and AFt were observed. The production of Ca(OH)_2_ remained at a relatively low level throughout the hydration reaction, and the presence of the peak of AFt in the XRD pattern of 3 d indicated that it was an early hydration product, the production of which facilitated a rapid increase in the early strength. In the XRD pattern, the main peak of C-S-H gels (PDF#34-0002) is very close to the main peak of calcite (PDF#05-0586), which is located at 29.405°. Because the samples are exposed to air during the curing process, the Ca^2+^ contained therein can react with CO₂ in the air through a carbonation reaction to produce CaCO_3_. Moreover, the Na^+^ contained therein can combine with CO₂ to generate Na₂CO_3_. Ultimately, a more stable product, CaCO_3_, is generated [17]. It can be further confirmed that the calcite is produced by the peaks located at 39.401° and 43.145°. However, considering that the portion of the samples exposed to air is relatively small as a proportion of the total, the amount of calcite is, therefore, low.

In the XRD patterns at 3 d, 7 d, and 28 d, the peak of the diaspore located at 22.2° is not observed in any sample except sample BF90C10. This indicates that AlO(OH) in the diaspore is gradually decomposed and dissolves in the slurry system due to the alkaline excitation of water glass [16,17]. Further, the comparison of Figure 9 with Figure 2b also reveals that the peaks of calcium sulfate dihydrate and calcium sulfate hemihydrate in the raw FGD almost disappear, indicating that CaSO_4_ in the FGD can be well-dissolved in the slurry system. The addition of FGD provides the red mud with a certain amount of active Ca^2+^ [15]. SO_4_^2−^ dissolved in the system becomes one of the raw materials involved in the hydration reaction. The SO_4_^2−^ ions facilitate the dissolution of Al^3+^ and Si^4+^ in BRM and cement, promoting the generation of hydrated gels and improving the compressive strength [52]. Thus, FGD is a solid-waste-type exciter in this study. As an alkaline exciter, water glass provides a large amount of active silica for red mud. It also promotes the dissolution rate of active Ca, Si, and Al from red mud and cement. Water glass and FGD together make up for the lack of activated Si and Ca in red mud, as mentioned above, and are able to produce strength.

It can be readily apparent from the XRD patterns of sample BF90C10 at 3 d, 7 d, and 28 d that the peaks of cancrinite [Na_6_Ca_2_Al_6_Si_6_O_24_(CO_3_)_2_·2H_2_O] (PDF#46-1332) are higher than the main peak of C-S-H gels. Observed in the raw red mud shown in Figure 2a, the presence of cancrinite means that most of it remains an inert component. A small number of C-S-H gels are generated in sample BF90C10, which indicates that there are few hydration products in this ratio and the strength formed is lower. Similarly, the peaks of hydration products in sample BF80C20 were elevated; yet, the total amount at 3 d and 7 d remained low. This is macroscopically manifested by its low compressive strength. The C-S-H gel is the main hydration product that forms the strength of this material. As the content of BRM-FGD decreases, the highest main peak gradually increases and slightly shifts to the left, indicating that more C-S-H gels are generated. The increase in hydration products (C-S-H gels) and the enhancement in strength correspond to the test result of the uniaxial compressive strength of BF-C grouting materials.

As can be seen from all three patterns at different curing periods, the positions of several major peaks do not change, indicating that the types of the main hydration products do not change as the curing time increases. A comparison of the XRD images of sample BF70C30 at 3 d, 7 d, and 28 d in Figure 9a–c shows that the main peak of the hydration product C-S-H gel is already high at 3 d. This indicates that the early hydration reaction rate is accelerated. The generated C-S-H gels are combined with an appropriate proportion of the inert component cancrinite within the system to form a higher early strength. This is more easily observed in the microscopic morphology of BF-C samples. When the ratio of BRM: FGD: cement is 66.5:3.5:30 (i.e., sample BF70C30), it can effectively improve the early strength.

**Figure 10:**
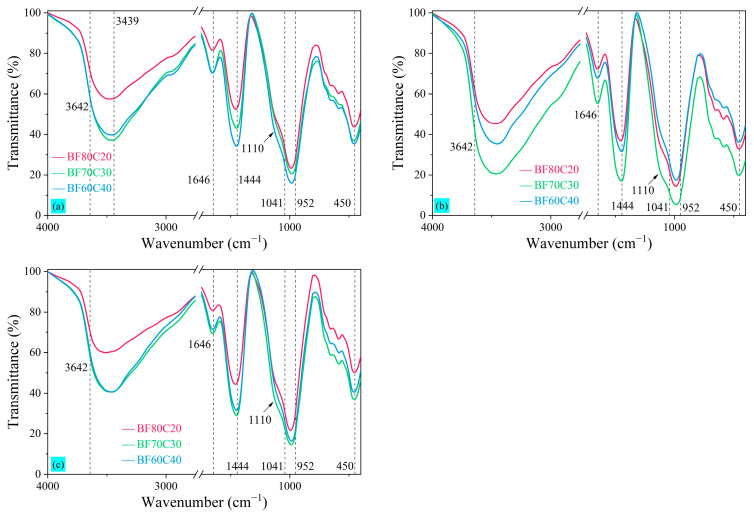
FTIR patterns of BF-C grouting materials at (**a**) 3 d, (**b**) 7 d, and (**c**) 28 d.

The microscopic compositions of the BF-C samples at 3 d, 7 d, and 28 d for the three ratios were further analyzed by observing the FTIR patterns, as shown in Figure 10. It can be seen that, ignoring subtle fluctuations, the spectra showed an analogous absorption peak variation. The absorption peak at around 1646 cm^−1^ is related to the bending vibration of the H–O–H bond in the interlayer water molecules contained in the samples [10]. The band around 3642 cm^−1^ was associated with the stretching vibration of Ca—OH in Ca(OH)_2_, and the band around 3439 cm^−1^ was related to the stretching vibration of Al–OH of Aft and also the absorption peaks of the O–H band, which indicated that a small amount of Ca(OH)_2_ and AFt was contained in the hydration reaction of the samples [48]. The main band of the asymmetric stretching vibrations of the Si–O bond (Si–O stretching vibrations typical of C-S-H gels) is located around 1041 cm^−1^, and its peak is shifted to lower frequencies because the system has a higher alkalinity [53]. Thus, the FTIR bonds at 3 d, 7 d, and 28 d are located around 982 cm^−1^ in this study. The peak at 450 cm^−1^ is also typical of C-S-H gels [3,53]. The appearance of the peak at 1110 cm^−1^ is because of the involvement of Ca^2+^ in the FGD, allowing more C-S-H and N-C-A-S-H to be produced [15]. Furthermore, combined with the calcite in the XRD pattern of Figure 9, the wide band around 1444 cm^−1^ is the stretching vibration peak of O–C–O in the carbonate group CO_3_^2−^, which is related to the CaCO_3_ generated by the carbonation reaction of CO_2_ in the air [3,10,53].

The FTIR patterns at 3 d in Figure 10a show that the amount of C-S-H gel increases gradually as the content of BRM-FGD decreases. However, the amount of C-S-H gel generated in sample BF70C30 is higher than that of BF60C40 at 7 d in Figure 10b. They are almost equal at 28 d in Figure 10c. This indicates that the hydration reaction in sample BF70C30 is accelerated and advanced during the first 7 days, and a higher amount of hydration products is generated at an early stage. Wang et al. [16] found that the polymerization degree of the chain structure formed mainly by C(N)-A-S-H and C-S-H increases first and then decreases with the increase in Ca/(Si + Al) from 0.75 to 1.03 and is the highest at 0.96. Therefore, the higher early compressive strength of sample BF70C30 could be due to the Ca/(Si + Al) ratio of 0.94 of the sample, and the synergistic effect between the raw materials within its slurry is the best. The wide band around 952~1014 cm^−1^ is related to the stretching vibration peaks of Si–O–T (T = Si, Al) in C-A-S-H or N-A-S-H [3,10]. At the beginning of the hydration reaction, C-S-H gels and other silicate products are generated in the system. As the hydration reaction continues, Al^3+^ dissolved from Al(OH)_3_ and AlO(OH) is able to replace some Si^4+^ on the [SiO_4_]^4−^ tetrahedra in the silicate to form C(N)-A-S-H. However, the peak of C(N)-A-S-H is not observed in the XRD pattern because its characterization is difficult to determine by XRD analysis [10]. Its presence can be observed in the SEM images.

### 3.4. Microscopic Morphology of BF-C

**Figure 11:**
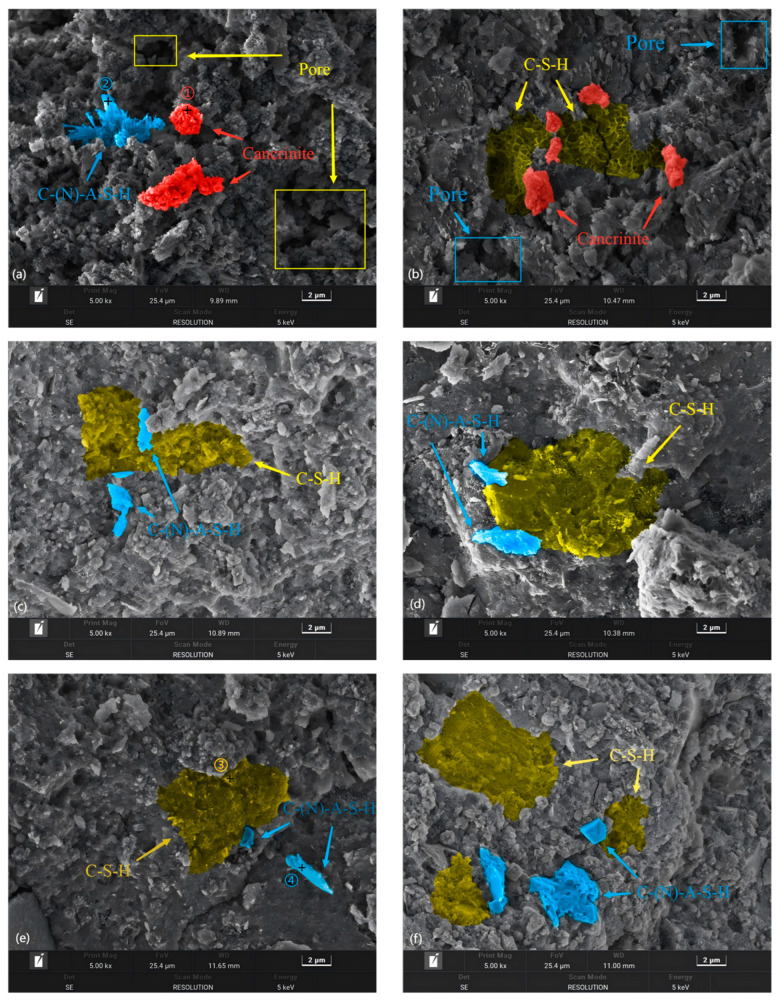
SEM of BF-C grouting materials: (**a**) BF90C10 at 3 d, (**b**) BF80C20 at 3 d, (**c**) BF70C30 at 3 d, (**d**) BF60C40 at 3 d, (**e**) BF70C30 at 7 d, and (**f**) BF70C30 at 28 d.

**Figure 12:**
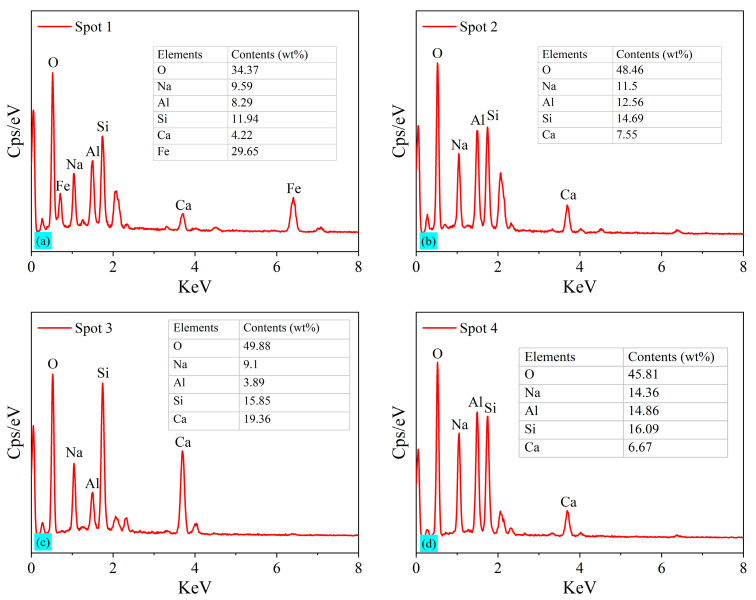
EDS of the spots marked in SEM images of BF-C grouting materials: (**a**) Spot 1, (**b**) Spot 2, (**c**) Spot 3, and (**d**) Spot 4.

The SEM images of BF-C grouting samples at 3 d and sample BF70C30 at 7 d and 28 d are shown in Figure 11. Meanwhile, the EDS patterns of the spots marked in the SEM images are shown in Figure 12. Spot 1 in Figure 12a is pointed at an agglomerated particle where the main elements are O, Na, Al and a large amount of Fe. It is the inert component of cancrinite. Spot 3 in Figure 12c is pointed at a three-dimensional network structure where the main elements are O, Si, and Ca, which indicates the existence of C-S-H gels. Spot 2 in Figure 12b and Spot 4 in Figure 12d are on an irregular crystal, where the element compositions are mainly composed of O, Na, Al, Si and a small amount of Ca, suggesting that C(N)-A-S-H gels are formed. This is consistent with the results obtained from the FTIR analysis. The hydration products, C-S-H gels, exhibit a three-dimensional network structure with a small number of clustered crystals, which might be C(N)-A-S-H formed by the replacement of Si^4+^ on the [SiO_4_]^4−^ tetrahedra by Al^3+^ [10].

In Figure 11a,b, a large number of pores can be observed inside samples BF90C10 and BF80C20. Their microstructures generally appear loose. Moreover, the exposed cancrinite that does not participate in the hydration reaction can be observed inside them. C-S-H gels cannot be observed in BF90C10. There are C-S-H gels inside BF80C20, but the generated C-S-H gels are not enough to encapsulate all the cancrinite, fill all the pores, and extend their own three-dimensional network structure. This is the reason for the low strengths of BF90C10 and BF80C20.

It is obvious from Figure 11a–c that, as the content of BRM-FGD gradually approaches 70 wt%, the microstructure of the samples becomes denser. Pores are largely unobserved inside sample BF70C30. The C-S-H gels are well-encapsulated on cancrinite and their three-dimensional network structure is also dense. A small number of C(N)-A-S-H crystals are interspersed among the C-S-H gels, resulting in a denser microstructure of the sample BF70C30. The destruction of the dense structure requires more energy, which is reflected in the increase in compressive strength [14,16]. Therefore, sample BF70C30 has a high early compressive strength. In summary, from a microstructural perspective, BF70C30 also shows a better synergistic effect than other samples.

The complete hydration processes of sample BF70C30 at 3 d, 7 d, and 28 d are shown in Figure 11c,e,f. A comparison among the three images reveals that sample BF70C30 had already generated a substantial amount of hydration products such as C-S-H gels and C(N)-A-S-H at 3 d and formed a dense structure. However, the types of subsequent hydration products did not change with the increase in curing time, only increased in quantity. This result is consistent with the XRD test results in Figure 9.

## 4. Conclusions

(1) In this study, a grouting material was prepared using Bayer red mud (BRM), flue gas desulfurization gypsum (FGD), and cement. When the ratio of BRM: FGD: cement was 66.5:3.5:30, the fluidity of the slurry was 230 mm, and the compressive strengths at 3 d, 7 d, and 28 d were 8.94 MPa, 9.86 MPa, and 13.71 MPa, respectively.
(2) FGD supplies more Ca^2+^ and SO_4_^2−^ to the system. Ca^2+^ serves as the main raw material for the hydration reaction, which increases the quantity of the hydrated products. SO_4_^2−^ ions promote the dissolution of Al^3+^ and Si^4+^ in BRM, thereby enhancing the activity of BRM.
(3) The main hydration products of the BF-C grouting materials are C-S-H gel, calcite, and a small amount of C(N)-A-S-H. The C-S-H gel shows a dense three-dimensional network structure. The amount of its generation plays a decisive role in the increase of strength.
(4) The Ca/(Si + Al) ratio is regulated by the FGD and alkaline activator. An appropriate Ca/(Si + Al) ratio can enhance the synergistic effect among BRM, FGD, and cement. The formation of hydration products is affected by the Ca/(Si + Al) ratio, which macroscopically manifests as a strength enhancement.
(5) Compared with existing methods, this study achieves the large-scale utilization of red mud in a concise and low-cost approach, alleviating the landfill problem of red mud while extending its value chain and promoting sustainability. The prepared grouting material can be used for sealing rock fissures. The durability and corrosion resistance of the products of the grouting materials in the study needed to be addressed and verified over a longer period.

## Figures and Tables

**Table 1:** Physical properties of red mud.

Physical Properties	Parameters
Particle diameter	0.088~0.25 mm
pH value	12.1~13.0
Specific gravity	2.7~2.9
Unit weight	0.8~1.0 g/cm^3^

**Table 2:** Chemical compositions of the selected raw materials (wt%).

Type	SiO_2_	Al_2_O_3_	Fe_2_O_3_	Na_2_O	CaO	MgO	K_2_O	TiO_2_	SO_3_	LOI
BRM	20.94	26.32	16.63	8.84	10.46	0.74	0.97	3.57	0.58	11.08
FGD	2.39	0.84	0.37	0.10	39.02	0.78	0.16	0.03	55.14	11.22
Cement	17.51	5.33	2.75	0.17	52.54	3.71	0.70	0.33	2.99	4.34

**Table 3:** Mixture proportions of the materials prepared.

Group	BRM /g	FGD /g	Cement /g	Alkaline Activator /g	Water Reducer /g	Water-Solid Ratio/wt%	Ca/(Si + Al) Ratio
BF100C0	95	5	0	14	1	0.5	0.42
BF90C10	85.5	4.5	10	14	1	0.5	0.57
BF80C20	76	4	20	14	1	0.5	0.75
BF70C30	66.5	3.5	30	14	1	0.5	0.94
BF60C40	57	3	40	14	1	0.5	1.14
BF0C100	0	0	100	14	1	0.5	-

**Table 4:** Relative humidity (RH) obtained at 30 °C with the saturated salt-water solutions used [44].

Salt	LiCl·H_2_O	MgCl_2_·H_2_O	NaBr	NaCl	K_2_SO_4_
RH (%)	11.28 ± 0.24	32.14 ± 0.14	50.00 ± 0.24	75.09 ± 0.11	97.00 ± 0.40

**Table 5:** Active element contents of the selected BRM and FGD raw materials (%).

Type	Si	Al	Fe	Na	Ca	Mg	K	Ti
BRM	15.02	6.86	9.19	6.28	1.75	0.23	0.74	1.85
FGD	2.05	0.27	0.26	0.06	23.38	0.51	0.12	0.02

**Table 6:** Compressive strength and increment of the samples (MPa).

	BF100C0		BF90C10		BF80C20		BF70C30		BF60C40	
Compressive strength at 3 d	0.23		0.54		2.40		8.94		10.06	
Strength increment at 3 d		0.31		1.86		6.54		1.12		
Compressive strength at 7 d	0.3		0.67		4.52		9.86		12.94	
Strength increment at 7 d		0.37		3.85		5.34		3.08		
Compressive strength at 28 d	0.53		3.08		5.41		13.71		17.04	
Strength increment at 28 d		2.55		2.33		8.30		3.33		

## Data Availability

The original contributions presented in this study are included in the article. Further inquiries can be directed to the corresponding author.
